# Commentary: Video games and stress: how stress appraisals and game content affect cardiovascular and emotion outcomes

**DOI:** 10.3389/fpsyg.2023.1191123

**Published:** 2023-06-15

**Authors:** Muhammad Rashid, Justin Szymczak, Nirmay Shah, Nicholas Lum, Michael Wong

**Affiliations:** ^1^Bachelor of Health Sciences Program, McMaster University, Hamilton, ON, Canada; ^2^Department of Psychiatry and Behavioural Neurosciences, McMaster University, Hamilton, ON, Canada

**Keywords:** Flow Theory, heart rate variability, stress, stress appraisal, video game, blood pressure, emotion

## 1. Introduction

In their study, Porter and Goolkasian (2019) explored whether physiological and psychological stress response to playing video games depends on how one appraises a stressful situation. Based on previous literature, the authors predicted that threat appraisals (inducing anxiety) would lead to increased blood pressure and higher negative emotion ratings relative to challenge appraisals (inducing excitement). To test this, Porter and Goolkasian compared the effects of threat and challenge appraisal when participants played the fighting-game Mortal Kombat (MK) and the puzzle-game Tetris. Threat appraisal was induced through difficult performance-based instructions during gameplay (e.g., time constraints), whereas challenge appraisal was induced through encouraging instructions (e.g., verbal reinforcement). As predicted, threat appraisal increased negative emotion ratings relative to challenge appraisal. However, contrary to predictions, blood pressure did not differ; the authors concluded this indicates that video games lack the evaluative stressors, such as public speaking, that are important for inducing stress.

Although the authors' conclusion is possible, we suspect this discrepancy may be caused by their study design, which did not carefully consider individual variability in skill level relative to the game's difficulty. Previous literature suggests one's appraisal of a stressor depends on the perceived demands of a task (Lazarus and Folkman, 1984; Blascovich et al., 1999). Although the authors frame their study after the Biopsychosocial Model of Challenge and Threat (Blascovich and Tomaka, 1996), we propose that Flow Theory may provide further insight into their findings (Csikszentmihalyi, 1990). Thus, our aim in this commentary is to examine the findings of Porter and Goolkasian within the framework of Flow Theory.

## 2. Variability in skill and difficulty levels

According to Flow Theory, proposed by Csikszentmihalyi (1990), different combinations of participant skill level and perceived difficulty of a task can lead to various states, from apathy and anxiety to relaxation and flow. People experience flow state when a task optimally matches their skill level and challenges them, deeply involving them in an enjoyable, focused, and motivating experience. Csikszentmihalyi contrasts this state with apathy, when the skill and challenge levels related to a task are both low, leading to no interest or concern for the task. Thus, the inability to get into flow state during a task can result in different emotional outcomes, depending on the skill and difficulty levels (see Csikszentmihalyi, 1990 for an in-depth explanation of Flow Theory).

Although Porter and Goolkasian (2019) made efforts to perform manipulation checks, they did not account for individual variability in skill level relative to the difficulty setting of each game. They predetermined the difficulty of the games based on the performance of only 10 pre-test participants. When the 148 participants self-reported their perceived difficulty post-test, their mean responses had a relatively large standard deviation. Based on Flow Theory, this suggests that participants who felt appropriately challenged, given their skill level, might have been in a state of flow, while those who felt the task was too difficult might have been in a state of anxiety (Csikszentmihalyi, 1990). Thus, averaging responses across individuals whose skill levels varied could have minimized an otherwise larger difference in stress outcomes between the two appraisal groups. Additionally, while the authors used self-reported measures to assess participants for general video game experience, they did not assess their experience specifically with MK or Tetris. Different genres and game mechanics have differential effects on the brain and require different behavioral responses; thus, skill levels in one game genre may not generalize to another (Mondéjar et al., 2016). In other words, some participants may be more skilled in MK or Tetris than others and more equipped for the pre-determined difficulty level and threat conditions. For more accurate correlations between instructions and stress outcomes, it is important to adjust each game's difficulty based on each participant's individual performance and take into consideration individual experiences with specific games (or game mechanics) before introducing threat and challenge appraisal instructions.

## 3. Casual video games and difficulty level

While the results of playing MK were generally consistent with the literature, that is, playing fighting games increased stress, the results of playing Tetris were not. Contrary to the literature, the authors did not find that playing a casual video game such as Tetris reduced physiological and psychological stress among their participants (Russoniello et al., 2009; Pine et al., 2020; Desai et al., 2021). The authors attribute this discrepancy to self-determination theory; however, we offer a complementary explanation using Flow Theory. On average, participants rated the difficulty of playing Tetris as 3.73 and 4.51 on a 5-point Likert scale in the challenge and threat conditions, respectively. Therefore, the difficulty setting of Tetris may have been too high relative to the participants' skill level and led them to states of anxiety or worry. In contrast to Porter and Goolkasian's study, other studies investigating the effects of playing casual video games on stress did not set a difficulty level for participants (for review see Pine et al., 2020). Participants played the games at their leisure for a set duration, which according to Flow Theory, could have led to a state of relaxation or control (Csikszentmihalyi, 1990).

## 4. Discussion

Given the intersection of skill level and perceived demand (or challenge), future studies would benefit from stratifying participants based on skill levels to minimize variability. Variability can be further minimized by implementing a repeated-measures design in which the same participants undergo both the threat and challenge appraisal conditions; a repeated-measures design may be especially important to consider given the individual nature of stress appraisal. Lastly, studies of this type may benefit from considering Flow Theory in their study design to provide a framework for understanding the intersection of skill and challenge (Csikszentmihalyi, 1990; Michailidis et al., 2018). Nevertheless, the findings of Porter and Goolkasian (2019) provide important insight into the individual nature of stress and the complexities related to stress research.

## Author contributions

All authors listed have made a substantial, direct, and intellectual contribution to the work and approved it for publication.
